# Reimbursement versus effort in medical physics practice in radiation oncology

**DOI:** 10.1120/jacmp.v4i2.2533

**Published:** 2003-03-01

**Authors:** Michael G. Herman, Michael D. Mills, Michael T. Gillin

**Affiliations:** ^1^ Division of Radiation Oncology Mayo Clinic 200 First St. SW Rochester Minnesota 55905; ^2^ Radiation Oncology Department James Graham Brown Cancer Center 529 S. Jackson St. Louisville Kentucky 40202; ^3^ Department of Radiation Physics M.D. Anderson Cancer Center Box 94, 1515 Holcombe Blvd. Houston Texas 77030

**Keywords:** medical physics costs, ambulatory payment classification, radiation oncology

## Abstract

The changes in health care reimbursement have the potential to affect the availability of quality medical physicist service in patient care. A survey was conducted by the AAPM Professional Council and the ACMP to collect cost information for special medical physics consultation, CPT4‐77370 and continuing medical physics, CPT4‐77336. The data collected from the survey was compared to current reimbursement schemes for a number of special procedures. Under varying reimbursement schemes, the costs of the medical physics services provided cannot be recaptured by the institution. It remains important for medical physicists to assess our utilization of resources and allocation to each of the services we provide and to understand the implications of policy changes at the federal and local levels.

PACS number(s): 87.90.+y, 87.53.–j

## INTRODUCTION

The cost of providing medical physics services is not clearly related to reimbursement for these services. The lack of data to substantiate the value and actual costs of medical physics services allows for reimbursement levels to be based on other data, or no data at all. This can lead to potentially insufficient and erroneous reimbursement models, especially as the Hospital Outpatient Prospective Payment System is based upon the costs of providing services.

To impact the Centers for Medicare and Medicaid Services (CMS), formerly Health Care Financing Administration (HCFA) process, it is necessary to collect and report quantitative data on the costs associated with providing quality medical physics services. In 1995, the Abt survey was conducted by the American College of Medical Physics (ACMP) and the American Association of Physicists in Medicine (AAPM), providing a basis of quantitative data^1^ for medical physics work effort. In addition, the ACMP commissioned a survey of physics resources for radiation oncology special procedures that provided benchmark values for the effort required to commission and sustain advanced procedures.^2^ Recent information demonstrates the importance of constructing a financial model for resource utilization built on quantitative data, specifically for medical physics.^3^ Additional cost information for medical physics procedures is necessary to provide medical physics administration with appropriate information to determine financially viable resource models. To that end, in 2001, a survey was conducted by the AAPM Professional Council and the ACMP to specifically address the cost of medical physics services for Current Procedural Terminology (CPT) 77336 Continuing Medical Physics and CPT 77370 Special Medical Physics Consultation. The primary goal was to build on the data collected in the earlier Abt survey and the special procedures resources survey in light of the new hospital outpatient prospective payment system (HOPPS) and ambulatory payment classification (APC) guidelines being published. This will allow comparisons of cost of effort versus reimbursement for different reimbursement mechanisms.

The regulations with respect to allowable billing for Medicare and Medicaid patients are very complex and change on a regular basis. Some of the statements contained in this paper represent the author's understanding of these rules at a specific point in time. The reader is advised to consult with their billing experts relative to specific details of billing for medical physics services. The authors make no implication about the relative value of medical physics compensation in this manuscript, rather compare cost with reimbursement values based on currently available information.

## METHODS AND MATERIALS

The survey in Appendix A was sent out in February 2001 to directors of (Therapy) medical physics departments and groups. The survey asked whether the medical physics group responding was an academic practice or a private/community hospital setting. The survey requested hourly wage ranges for each medical physicist and the institutional overhead rate to calculate costs of providing services. Each respondent stated the number of hours a qualified medical physicist (QMP) spent on CPT 77336 per week for patient specific as well as any additional related effort. CPT 77336 Continuing radiation physics includes assessment of treatment parameters, quality assurance of dose delivery, and review of patient treatment documentation in support of the radiation oncologist, reported per week of therapy. Procedure code 77336 is specific to the review of the weekly radiation treatment plan. This service ensures that the treatment administered conforms to the specifications of the prescribing physician. This service includes a documented review of the patient's treatment chart and record to verify that the patient received the prescribed radiation dosage, appropriate positioning and beam orientation, and radiation safety. This procedure is reported weekly (once every consecutive five treatments delivered), a frequency that would match the weekly radiation treatments billed.

As defined in this survey and in the previous Abt work, CPT 77336 includes all of the following tasks:

- Reviewing the patient case in initial presentation
- Performing weekly chart checks of all charting, diagnostic studies, port films and patient calculations
- Reviewing charts with other members of the patient management team
- Viewing patient positioning and machine setup
- Researching treatment scheme
- Performing the final chart check and validation of the treatment

77336 does not include machine or program tasks such as commissioning and ongoing quality assurance. It is also assumed that 77370 is not billed for the above listed services.

The survey also requested the number of hours of QMP effort committed to various special procedures, where the CPT 77370 was reported. CPT 77370 Special Medical Radiation Physics Consultation is used for consultative purposes when a problem or special situation arises during radiation therapy. This code requires a written request by the physician and a detailed written report describing the problem to be reviewed. Typical uses of CPT 77370 include the following services:

- total skin electrons (TSE)
- total body irradiation (TBI)
- remote afterloading HDR brachytherapy (HDR)
- low dose rate (LDR) brachytherapy
- prostate implants, intra‐operative radiation therapy (IORT)
- stereotactic radiosurgery, stereotactic radiotherapy
- electron arcs
- patients with pacemakers close to the treatment field
- 3D treatment planning*
- special calculations
- review of patient on treatment couch
- review of patient simulation procedure
- stereotactic brachytherapy
- intravascular brachytherapy
- intensity modulated radiation therapy (IMRT)*

Three‐dimensional treatment planning and IMRT are marked with an asterisk, as any effort involved in the use of the CPT 77370 related to these procedures is beyond and above the treatment planning effort itself. Special Medical Physics Consultation would be used in addition to either 77295 or 77301, and billed on a separate date, for additional effort such as dosimetric validation based on extensive measurements specifically for a given patient. Information was requested on QMP hours per patient procedure, total commissioning hours expended for that procedure, and annual hours of ongoing nonpatient effort to support the procedure. Many of these procedures were considered in the medical physics resource survey. For those procedures where data exists, comparisons with the current survey were made.

Hourly wages and overhead were used to calculate cost to the institution per hour of medical physics service. A value for the 2001 average hourly wage was extrapolated from the AAPM salary survey for comparison. Medical physicist cost data was adjusted to 2002 by using a 3% increase for comparison with reimbursement levels in 2002.

Costs for CPT 77336 were derived from the reported hours per week from the survey. Additional costs, including any additional effort in support of the 77336, were also based on the hourly wage values. Costs were annualized for comparison with data from the Abt study and for comparison with different reimbursement rates. The survey costs were annualized by calculating the total FTE committed to CPT 77336. Thus for the current survey, regardless of the number of CPT 77336 procedures performed, a constant cost to the institution is reported. Cost and reimbursement values were calculated for 500, 1000, and 1500 procedures per year, to present a range of data, as the actual time required to perform this procedure varies between clinics. Also calculated is the cost based on the Abt survey value of 1.5 hours per CPT 77336.

Costs for the CPT 77370 activities were calculated on a per patient basis from the average hours per patient procedure, for the average total hours of commissioning, and for the average total annual ongoing effort of the QMP. The costs calculated for 77336 and 77370 are compared to reimbursement values from 2002 current procedural code for (CPT4) (Medicare and 75th percentile non‐Medicare), 2002 APC, and for comparison, the original 2001 APC values assigned to these medical physics services.

## CURRENT REIMBURSEMENT SCHEMES

Table I demonstrates reimbursement rates, based on the 2002 CPT4 values both for Medicare and the 75th percentile for non‐Medicare billings for each medical physics service.^4^ Also listed are the 2002 values for APC 304 (representing CPT 77336) and APC 305 (representing CPT 77370).^5^ In addition, also listed is the value of the APC for category 311, which in 2001 had both 77336 and 77370 grouped together at one rate. Note that Medicare CPT4 values are representative and may have regional correction applied to them for a specific geographic region.

**Table I:** Reimbursement schemes for CPT 77336 and 77370.

		CPT4		APC 304 level I	APC 305 level II radiation treatment	APC 0311 radiation physics services
Service		Non‐Medicare 75%	Medicare			
77336	Continuing radiation physics (weekly)	$343	$120	$91.52	—	$74.11
77370	Special medical physics consultation	$446	$140	—	$223.70	$74.11

## RESULTS

Thirty complete surveys were returned and analyzed. Eleven were from academic medical centers and the remainder from community hospitals and private practice. The average hourly wage for a qualified medical physicist (QMP), adjusted to 2002, was $59.03 (median $58.7), with a minimum of $30.00 and a maximum of $90.00. This value compares very well with the result extrapolated from the AAPM salary survey 1998–2001 to 2002 values for physicists who practice primarily in radiation oncology of $58.90. The average institutional overhead rate was 28%, which implies an hourly total cost of a QMP to the institution of $75.66.

The QMP spent an average of 13 (median 12) h per week performing 77336 (as defined in the survey), with an additional 6 (median 4) h of time devoted to tasks in support of this procedure. Ranges were 1.25–50 h. This amounts to 988 h per year devoted to the service of 77336 for a QMP.

The 1995 Abt data indicates that 3.5 FTE are committed to performing 77336 for 800 patients per year. With an average of 4.5 77336 services per treatment course, then each QMP would perform 1028 instances of the 77336 per year. Therefore, the average time per 77336 is then 988/1028=0.96 hour or approximately 1 h. The total cost to the institution to provide this service, based on the average salary cost would be $74 783 (based on 988 hours out of a 2080 hour year). The Abt survey indicated that the 77336 median value was 1.5 h.

Table II indicates the annual reimbursement for CPT and APC schemes for a minimum and maximum number of 77336 services performed by a QMP. Under certain circumstances, neither

**Table II:** Reimbursement values for various schemes.

Billed 77336 annually	Reimbursement				Cost	
	CPT4, Non‐Medicare	CPT4, Medicare	APC311	APC304	Current survey	Abt survey
500	$171 500	$60 000	$37 055	$45 760	$74 783	$56 748
1000	$343 000	$120 000	$74 110	$91 520	$74 783	$113 495
1500	$514 500	$180 000	$111 165	$137 280	$74 783	$170 243

APC may cover the institutional expense incurred to provide the 77336 service. The cost is calculated from the average salary cost and benefits previously defined. The model does not consider the fact that 100% of billed services are not collected.

The cost to provide the service, based on the Abt survey data, which indicated that the 77336 accounted for 1.5 hours of QMP time, and the hourly total QMP cost is also indicated in the table. Under these conditions, the HOPPS APC reimbursement schedule would never cover the cost of providing 77336 services.

Table III summarizes the results of the current survey with respect to QMP hours (Column 2) required to provide 77370 for each of the services listed in Column 1. For comparison purposes, the result from the ACMP *survey of physics resources for radiation oncology special procedures* ^2^ is also listed where the data exist (Column 3). In most cases, the current survey data agrees well with the resource survey data. The associated cost to the institution, based on cost tabulated earlier, is indicated in Column 4, which can be directly compared to the reimbursements in CPT and APC (Table I). Finally, the number of responses are given in Column 5. It should be noted that the cost values indicated here only represent patient procedural time and exclude commissioning and continuing QA.

**Table III:** QMP hours per special procedure for current survey, resource survey, and associated costs per patient procedure.

Procedure Current survey	QMP hours per patient		Cost this survey	Number of responses
	Current survey	Resource survey		
Total skin electrons	9.50	9	$719	10
Total body irradiation	5.03	9	$381	16
Remote afterloaded HDR brachytherapy	2.72	8	$206	25
LDR brachytherapy	3.25	na	$246	23
Prostate implants	6.76	na	$511	25
Intra‐operative RT	4.57	6	$346	7
Radiosurgery	5.34	11	$404	16
Stereotactic radiotherapy	6.68	na	$505	14
Electron arc	11.75	9	$889	4
Pacemaker treatment	1.82	na	$137	19
3D treatment planning	3.51	5.1	$265	29
Special calculations	2.26	na	$171	27
Review of patient on Tx couch	0.58	na	$44	27
Review of patient simulation	0.80	na	$61	27
Stereotactic brachytherapy	11.00	15	$832	4
Intravascular brachytherapy	3.15	na	$239	13
Intensity modulated RT	12.03	na	$910	15

**Figure 1:**
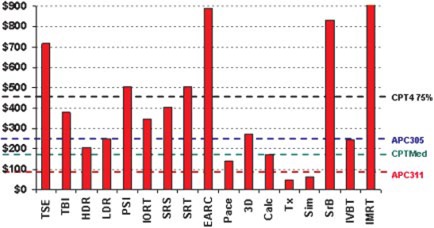
(Color) Costs of providing 77370 for various procedures compared to reimbursement schemes.

For comparison, the 77370 data are shown graphically in Fig. 1. The calculated costs to perform the services for each procedure are shown as bars and the reimbursement levels for CPT4 Non‐Medicare (75%), CPT4 Medicare, APC305 and APC311 are indicated as horizontal lines. These data are *exclusive* of commissioning and QA effort.

Table IV exhibits the cost of initial commissioning, and Table V ongoing quality assurance for each of the special procedures. These data represent additional costs to the institution, none of which is captured in the CPT 77370 billing. The commissioning and QA for routine external beam use of the linac may be included in the CPT 77300–77315 codes. It is not clear how much of the special procedures work (if any) is included in these codes, as the number of these procedures used at different clinics varies greatly.

**Table IV:** Hours and costs for initial commissioning of special procedures.

Procedure	Total hours commissioning:		Cost this survey	Responses
	Current survey	Resource survey		
Total skin electrons	100.89	105	$7 634	9
Total body irradiation	70.00	85	$5 296	13
Remote afterloaded HDR brachytherapy	55.95	170	$4 233	20
LDR brachytherapy	24.31	na	$1 840	16
Prostate implants	70.88	na	$5 363	17
Intra‐operative RT	129.33	225	$9 786	6
Radiosurgery	137.50	200	$10 404	12
Stereotactic radiotherapy	110.00	na	$8 323	10
Electron arc	126.67	200	$9 584	3
Pacemaker treatment	26.58	na	$2 011	6
3D treatment planning	213.10	425	$16 124	21
Special calculations	26.13	na	$1 977	8
Review of patient on treatment couch	37.83	na	$2 863	6
Review of patient simulation	26.57	na	$2 010	7
Stereotactic brachytherapy	113.33	100	$8 575	3
Intravascular brachytherapy	61.70	na	$4 668	10
Intensity modulated RT	302.50	na	$22 888	12

**Table V:** Hours and costs of QMP ongoing effort for special procedures (current survey).

Procedure	Hours ongoing effort (QA)	Cost/year	Responses
Total skin electrons	9.75	$738	8
Total body irradiation	21.10	$1 596	10
Remote afterloaded HDR	69.19	$5 235	21
LDR brachytherapy	15.26	$1 155	17
Prostate implants	41.79	$3 162	19
Intra‐operative RT	36.40	$2 754	5
Radiosurgery	62.50	$4 729	12
Stereotactic radiotherapy	46.30	$3 503	10
Electron arc	28.00	$2 119	3
Pacemaker treatment	8.30	$628	10
3D treatment planning	124.55	$9 424	22
Special calculations	10.33	$782	9
Review of patient on treatment couch	104.50	$7 907	8
Review of patient simulation	89.38	$6 762	8
Stereotactic brachytherapy	15.67	$1 185	3
Intravascular brachytherapy	45.82	$3 467	11
Intensity modulated RT	133.00	$10 063	13

## DISCUSSION

Medical physics procedure cost data is presented and compared to various reimbursement schemes. Costs for CPT 77336 were such that under certain circumstances, costs for providing this service are not recovered by the institution. If one uses the Abt data for this procedure, the schemes are even less favorable, due to the larger value reported for QMP time for this procedure. The apparent difference between the Abt time (1.5 h) and this survey (1.0 h) may be due to the assumption in this survey that a work week consists of 40 h. Also it should be noted that APC311 was eliminated in 2002 and CPT 77336 was moved to APC 304, which provided a significant increase in the reimbursement rate.

For CPT 77370, data is presented that reflects the *labor QMP* cost per patient procedure to provide special medical physics consultation. These range from $44 for reviewing patient setup and position during treatment to over $800 for IMRT, total skin, electron arc, and stereotactic

brachytherapy. The reimbursements for CPT 77370 are fixed per reimbursement scheme and range from $140 for Medicare to over $440 for non‐Medicare coverage. Clearly some procedures, being much more intensive than others, are not reimbursed at or above cost. Further, whether the commissioning and quality assurance of special procedures is reimbursed in any scheme is unclear.

Employers should identify multiple revenue streams upon which to base the salaries of medical physicists. These revenue streams should include billable services for patient care, such as 77336 and 77370. They should also be based upon general overhead charges for non‐patient specific services, which can range from general radiation safety to annual review of a treatment device. The physics time required to commission and support treatment planning systems and special procedures should also be included in establishing an overhead charge. One important weakness, as hospital outpatient facilities adjust to the APC's, is the establishment of the actual costs of providing all services, including those provided by the medical physicist. The data provided here should help physicists and their institutions in establishing their own cost environment.

Through vigilant monitoring and direct proactive efforts such as establishing the actual costs of providing medical physics services in radiation oncology, improvements that benefit all practicing medical physicists can be made. Working together with other professional groups, including the American College of Radiology and the American Society for Therapeutic Radiology and Oncology, is a powerful mechanism to affect these patterns in a positive manner.

This survey is based upon 30 responses from selected institutions. The data presented here may not accurately reflect the cost to provide medical physics services at a specific institution nor should this data be generalized as average costs at all institutions. An updated Abt survey was commissioned by the ACMP and the AAPM in 2002 and is currently being analyzed.

Detailed information on APC values and CMS activities on reimbursement is available in the published Federal Register ((http://www.archives.gov/federal‐register/index.html). In addition the ASTRO/ACR Joint Economics Committee has generated a user's guide to radiation oncology coding.^6^

The changes in health care reimbursement have the potential to affect the availability of quality medical physicist service in patient care. It is in our best interest to continue to monitor these conditions and to understand the implications of policy changes at the federal and local levels. It is also prudent to accurately assess our utilization of resources and allocation to each of the services we provide. Under varying reimbursement schemes, the costs of the medical physics services provided cannot be captured by the institution. The inability to capture the costs of providing medical physics services may have a negative impact on patient care, if the result is diminished accuracy and safety in radiation oncology.

## APPENDIX A

ACMP/AAPM Medical Physics Service Cost Survey

Dear,

February 28, 2001

This brief survey is being sent to you to gather critical resource information to substantiate the cost of providing medical physics services. The final HOPPS/HCFA rule diminishes the Medicare reimbursement for 77336 (continuing medical physics services; question 3 below) and 77370 (special medical physics consultation; question 4 below) to $64.00. Quantitative data on resource requirements and costs to provide these services is needed to demonstrate to HCFA the true cost of delivering these services.

The ACMP and AAPM commissioned the initial Abt survey to measure work performed by the qualified medical physicist (QMP). The Abt survey indicated that the median procedural time required to provide services was 1.5 hours for a 77336 and 4.0 hours for a 77370. This included the time for activities that were directly related to the performance of the procedure only. This did not include time for non‐procedural tasks such as periodic maintenance or quality assurance. The Abt report is available on the ACMP web site (www.acmp.org/publications.html). While AAPM and ACMP will conduct a second comprehensive survey with Abt Associates over the next 1–2 years, initial data on these two important Medical Physics charges is needed now. Your responses will be held anonymously and in complete confidence. Please return your survey in the enclosed self‐addressed envelope to ACMP headquarters for processing as soon as possible and preferably in the next 3 weeks. Questions may be addressed to acmp@acmp.org, or any of us.

Thank you for contributing to this important effort in support of Medical Physics,

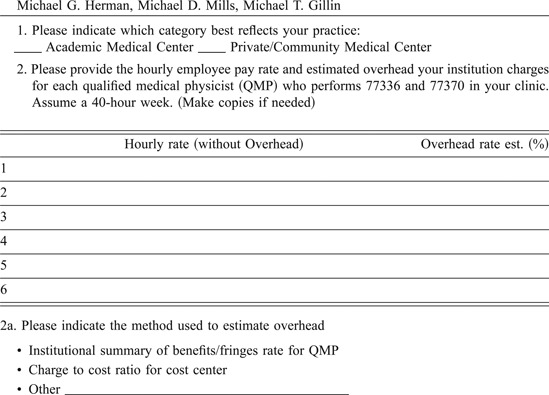

2a. Please indicate the method used to estimate overhead

- Institutional summary of benefits/fringes rate for QMP
- Charge to cost ratio for cost center
- Other_________________________________________

3. The Abt survey defined the duties associated with 77336 to include:

- Performing an initial patient case review
- Researching treatment scheme (assuming CPT 77370 is not billed)
- Viewing patient positioning and set‐up during simulation and at the treatment machine
- Performing weekly chart check of all charting, diagnostic studies, port films, and patient calculations.
- Reviewing charts with other members of patient management team (e.g. treatment planning conference)
- Performing final chart check and validation

What are the average hours per week spent by each FTE QMP for 77336 as defined above in your clinic?#x005F;____3a. If machine, device and Planning System QA/management are included in question 3 above what additional hours are spent by each FTE QMP per week?_________________

4. For each of the procedures listed below, please indicate the number of hours the QMP spends providing the billable 77370 service per patient procedure. It is assumed that a physician requests this service and the QMP generates a written report. For each item estimate the total initialcommissioning time and ongoing effort(QA) time for each procedure in hours per year:

**Table:** 

Service per patient	QMP hours per patient	Total hours Commissioning:	Yearly hours Ongoing Effort (QA)
Total Skin Electrons			
Total Body Irradiation			
Remote afterloaded HDR brachytherapy			
LDR brachytherapy			
Prostate Implants			
Intra‐operative RT			
Radiosurgery			
Stereotactic Radiotherapy			
Electron arc			
Pacemaker treatment			
3D treatment planning			
Special calculations			
Review of patient on treatment couch			
Review of patient simulation			
Stereotactic brachytherapy			
Intravascular brachytherapy			
Intensity Modulated RT			
Others			
